# Serotonin antidepressants and atrial fibrillation burden from cardiac implantable electronic devices

**DOI:** 10.1002/joa3.12948

**Published:** 2023-10-30

**Authors:** Youlin Koh, Cecilia Kwok, Aleksandr Voskoboinik, Jonathan M. Kalman, Michael Wong

**Affiliations:** ^1^ Department of Cardiology Western Health St Albans Victoria Australia; ^2^ Department of Cardiology Royal Melbourne Hospital Melbourne Victoria Australia; ^3^ Department of Cardiology Alfred Health Melbourne Victoria Australia

**Keywords:** antidepressant, atrial fibrillation, cardiac implantable electronic devices, pacemaker, selective serotonin reuptake inhibitors

## Abstract

**Objective:**

Depression and anxiety show a bidirectional relationship with atrial fibrillation (AF). Antidepressant use is associated with a reduction in the incidence of AF. However, no studies have examined the relationship between antidepressant use and AF burden (time in AF). This retrospective cohort study examined cardiac implantable device‐detected AF episodes and their relationship with antidepressant use, among other treatment factors.

**Methods:**

Consecutive patients from the Western Health Cardiology Department attending pacemaker checks between 2015 and 2021 were included. Patients with permanent AF were excluded, yielding 285 patients with no or paroxysmal AF, with a total of 772 patient encounters. Generalized estimating equations were used to model two processes: binary AF (present/absent) and the number of days in AF for patients with AF.

**Results:**

Each yearly increase with age was associated with an increase in the odds of developing AF (OR 1.03 [1.00–1.05], *p* = .027). Male gender conferred a reduction in AF incidence (OR 0.30 [0.13–0.68], *p* = .004). Digoxin use was associated with incident AF (OR 4.43 [1.07–18.4], *p* = .04). Sotalol and heart‐failure beta blocker use were associated with a decrease in AF burden (IRR 0.30 [0.12–0.78], *p* = .013 and 0.33 [0.14–0.81], *p* = .015). Selective serotonin reuptake inhibitor antidepressant use was associated with reduced AF burden (IRR 0.27 [0.09–0.81], *p* = .019), as was selective serotonin/noradrenaline reuptake inhibitor use (IRR 0.07 [0.03–0.15], *p* < .001).

**Conclusions:**

Older age, female gender and digoxin are associated with a higher odds of developing incident AF. Sotalol, heart failure beta blockers and serotonin‐based antidepressants are associated with reduced AF burden. Further prospective study into the effects of antidepressants on atrial arrhythmias is warranted.

## Graphical abstract

Depression and anxiety are both associated with incident atrial fibrillation. In our study examining atrial fibrillation burden as measured on cardiac implantable devices, serotonin‐based antidepressants were associated with a reduced AF burden, although they did not have an impact on the presence or absence of AF in the first instance.

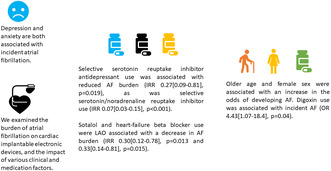

## 1 BACKGROUND

The incidence and prevalence of atrial fibrillation (AF) is increasing globally, contributing to increased hospitalization, healthcare utilization and costs, and limitations in quality of life.^1^, ^2^ Limitations in quality of life are in part contributed by anxious and depressive states, which are associated with increased AF symptom burden.^3^ The relationship between AF, depression and antidepressant use has been observed in multiple settings, including incident AF in both general and cardiac populations.^4^ On the other hand, the link between anxiety and incident AF has not been strongly demonstrated in population studies,^5^ but specific anxiety disorders such as panic disorder^6^ have been shown to have an association with incident AF. Negative psychological states outside of “traditional” depression or anxiety (e.g., vital exhaustion) have also been found to contribute to a higher risk of AF,^7^ suggesting a more general relationship between negative affective states and the risk of incident AF.

Depressive symptoms are also associated with AF recurrence in patients undergoing rhythm control strategies in the post‐cardioversion^8^ and post‐catheter ablation settings.^9^ Higher baseline anxiety scores also predict AF recurrence after catheter ablation.^9^ However, this relationship is also bidirectional, with marked improvements in psychological distress and suicidal ideation seen post atrial fibrillation ablation.^10^

Although antidepressant medications are used to treat both depression and anxiety, there is little data on their impact on AF burden. Despite demonstrated associations between depression and autonomic dysfunction in the form of altered heart rate variability (HRV),^11^ which may be the mechanistic link behind arrhythmia risk, there still exists clinical uncertainty around the role of antidepressants in this relationship, with authors postulating that antidepressants, rather than the underlying psychopathology, contribute to altered HRV.^12^

Our study aims to add to this field of research by analyzing the relationship between antidepressant exposure and AF outcomes, namely (1) the presence of AF (binary yes/no) and (2) AF burden (number of days in AF). A diagnosis of depression or anxiety, and other pharmacotherapies for AF, are examined as covariates of secondary interest.

## 2 METHODS

**FIGURE 1:**
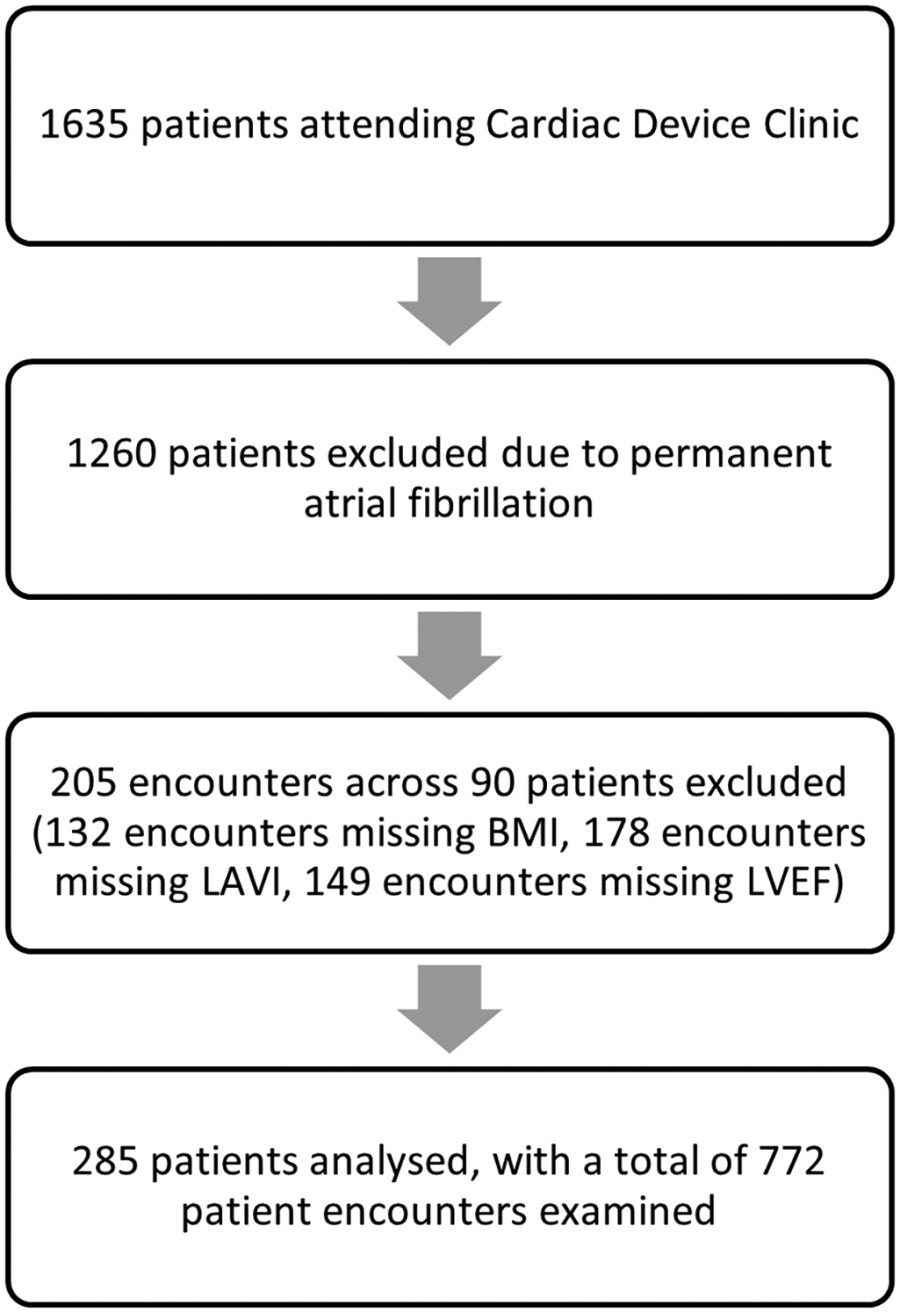
Flow diagram for patient selection. BMI, body mass index; LAVI, left atrial volume index; LVEF, left ventricular ejection fraction.

In this retrospective cohort study (Figure 1), consecutive patients over the years 2015–2021 from the Western Health Cardiac Device Clinic were examined. Of 1635 unique patients, 1260 were excluded from the study due to permanent AF. Ninety patients with missing variable information across 205 encounters were further excluded (complete case analysis). Two hundred and eighty‐five patients with no or paroxysmal AF were analyzed, with a total of 772 patient encounters across the 7‐year period.

Baseline characteristics such as age, sex, and body mass index (BMI) were obtained from hospital medical records. Diagnoses of depression and/or anxiety, left atrial volume index (LAVI), left ventricular ejection fraction (LVEF), as well as cardiac and psychiatric medication use, were obtained from the International Classification of Diseases 10 (ICD‐10) coded databases and hospital medical records. The follow‐up for each patient commences at the time of first clinic attendance in the period included in the study; data before this was not included in the model. Antidepressants examined included selective serotonin reuptake inhibitors (SSRIs, e.g., sertraline, citalopram, and fluoxetine), selective serotonin/noradrenaline reuptake inhibitors (SNRIs, e.g., venlafaxine, desvenlafaxine, and duloxetine), and tetracyclic antidepressants such as mirtazapine. In our population, rhythm control was undertaken with sotalol and amiodarone. Propafenone is not available in Australia, and flecainide was not prescribed in our cohort of elderly patients.

CIEDs measure AF burden as the time spent in AF across all of the days elapsed between checks. For example, 75 out of 150 days that has elapsed between checks is recorded as 50% AF burden. To obtain AF burden as a number of days, this percentage from each device check was multiplied by the number of days elapsed between checks. As patients undergo multiple checks during the lifetime of their device (usually every 6–12 months), each patient usually had more than one check. For each patient, the varying number of days of each check was accounted for by including the total number of days of each check as an offset variable. Data cleaning involved cross‐tabulation to examine for missing values and the removal of patients in permanent AF. All checks were manually adjudicated to ensure correct categorization of episodes as although largely accurate, misinterpretation of cardiac rhythm by CIEDs can still occur.

Means and standard deviations, or median and interquartile ranges for non‐normally distributed data, are presented for baseline characteristics between groups (antidepressant vs. no antidepressant exposure). Proportions were used for categorical variables. Univariate significance testing was carried out using Kruskal–Wallis and chi‐square tests. We then modelled the relationship between various independent variables and the dependent variable, the total number of days in AF.

The relationship between number of days in AF and various independent variables were modelled using the method of generalized estimating equations (GEE), due to repeated measurements (multiple checks per patient) leading to clustering of measurements. GEE, an extension of generalized linear models, models the average response of the population instead of a within‐subject covariance structure, using an empirical variance estimator (also known as the Huber‐White or sandwich estimator). It accounts for within‐subject or within‐cluster correlations. GEE assumes a working correlation structure that may differ from the true correlation structure, but is robust to misspecification of the working correlation.^13^

The data was analyzed with two separate models due to the possibility of differing effects of clinical factors on (1) the risk of incident AF and (2) the severity of AF, once present. The first analysis was based on AF absent/present, undertaken with a logistic regression within the GEE model. The second analysis was undertaken by specifying a negative binomial count distribution within the GEE model, restricting analysis to only patients who had recorded days of AF on their device checks. There are two resulting sets of coefficients, one for the binary part and one for the count part.

All statistical analyses were performed with Stata version 17.0 (StataCorp. 2021. Stata Statistical Software: Release 17. College Station, TX: StataCorp LLC).

## 3 RESULTS

### 3.1 Baseline characteristics

Characteristics of the 285 study participants are shown in Table 1. SSRIs were the most commonly used antidepressant across all 73 antidepressant use episodes, with 29 use episodes (39.7%). SNRIs constituted 22/73 use episodes (30.1%), and tetracyclic antidepressant (mirtazapine) use 18/73 episodes (24.7%). Not all patients who had a mental health diagnosis were taking an antidepressant; for example, 3% of patients with depression and 0.9% of patients with anxiety were not on any medication.

**TABLE 1:** Baseline characteristics of patients.

	No antidepressant (*n* = 249)	Antidepressant (*n* = 36)	*p*‐value
Age (median, IQR)	75 [62.0–82.0]	73 [67.0–84.0]	.317*
Male sex (%)	156 (62.7)	18 (50.0)	.146**
Depression (%)	8 (3.21)	13 (36.1)	<.001**
Anxiety (%)	2 (0.8)	5 (13.9)	<.001**
BMI kg/m^2^ (median, IQR)	27.8 [24.7–31.7]	30.3 [25.4–33.0]	.165**
LAVI ml/m^2^ (median, IQR)	33.5 [28.0–42.4]	34.6 [27.9–40.7]	.837**
LVEF (%) (mean, SD)	47 (13)	45 (12)	.371***
Amiodarone (%)	13 (5.2)	5 (13.9)	.046**
Sotalol (%)	9 (3.6)	1 (2.8)	.799**
Digoxin (%)	10 (4.2)	5 (13.9)	.013**
Beta blocker (%)			
1. Heart failure beta‐blocker	58 (23.3)	12 (33.3)	.097**
2. Non‐heart failure beta‐blocker	28 (11.2)	87 (19.4)	
AF present/absent	103 (31.2)	13 (29.6)	.832**
No. of days in AF	0 [0–0.84]	0 [0–0.88]	.723*
No. of days of follow‐up	252 [61–583]	285.5 [75–578]	.956*

Abbreviations: AF, atrial fibrillation; BMI, body mass index; IQR, interquartile range; LAVI, left atrial volume index; LVEF, left ventricular ejection fraction; SD, standard deviation.

* Kruskal–Wallis test.

** Chi‐square test.

*** *t*‐test.

There was no significant difference in BMI, LAVI or LVEF between groups. With regards to AF medications, there were significantly more patients in the antidepressant group taking amiodarone (13.9% vs. 5.2%, *p* = .046), and digoxin (13.9% vs. 4.2%, *p* = .013).

There was no significant differences in the presence of AF across antidepressant groups: 31.2% in the no antidepressant group had AF versus 29.6% in the antidepressant group, *p* = .832. Most patients with atrial fibrillation had a very low burden of AF, with a median of 0, IQR [0–0.84] days for the no antidepressant group and 0, IQR [0–0.88] days for the antidepressant group. The range of the total number of days in AF was wide, from 0 to 1149 days (data not shown). The number of days of follow up was not significantly different between groups.

### 3.2 Univariate regression

Results for univariate regression on variables related to AF burden are summarized in Table 2. For the binary portion (AF present/absent), each yearly increase in age was associated with a 3% increase in the odds of developing AF (OR 1.03 [95% CI 1.00–1.07], *p* = .007). Male sex was associated with a reduced odds of AF (OR 0.35 [0.17–0.69], *p* = .002) as compared to females. Depression, anxiety, and antidepressant use were not significantly associated with the odds of incident AF. Sotalol was associated with an increased risk of AF (OR 3.28 [1.06–10.1], *p* = .039), and digoxin use conferred an approximately 7.6‐fold increase in the odds of AF (OR 7.67 [2.21–26.6], *p* = 0.001).

**TABLE 2:** Univariate regression coefficients for independent variables affecting AF burden (unadjusted analysis).

Variable	Level	Odds ratio zero‐part (95% CI)	*p*‐value	Incidence rate ratio count part (95% CI)	*p*‐value
Antidepressant use	None SSRI SNRI Tetracyclic	1 0.82 [0.22–3.07] 0.77 [0.10–5.76] 2.68 [0.42–17.1]	— .777 .798 .298	1 .29 [0.09–0.95] .09 [0.07–0.13] 2.37 [0.45–12.6]	— .042 <.001 .309
Age (years)		1.03 [1.00–1.07]	.007	1.00 [0.96–1.03]	.865
Sex	Female Male	1 0.35 [0.17–0.69]	— .002	1 1.01 [0.48–2.13]	— .980
Depression	No Yes	1 0.98 [0.32–3.04]	— .979	1 1.04 [0.35–3.08]	— .07
Anxiety	No Yes	1 3.19 [0.61–16.7]	— .169	1 0.51 [0.13–1.88]	— .313
BMI		1 0.99 [0.94–1.05]	— .801	1 1.03 [0.98–1.09]	— .198
LAVI		1 1.00 [0.97–1.03]	— .988	1 1.03 [1.0.1–1.05]	— .003
LVEF		1 1.00 [0.98–1.02]	— .866	1 0.97 [0.96–0.99]	— .006
Amiodarone use	No Yes	1 0.82 [0.19–3.56]	— .787	1 0.73 [0.35–1.55]	— .416
Sotalol use	No Yes	1 3.28 [1.06–10.1]	— .039	1 0.22 [0.11–0.44]	— <.001
Digoxin use	No Yes	1 7.67 [2.21–26.6]	— .001	1 1.07 [0.53–2.16]	— .849
Beta blocker use	None Heart failure beta blocker General beta blocker	1 0.96 [0.44–2.11] 1.91 [0.29–5.30]	— .922 .209	1 1.03 [0.64–1.65] 0.94 [0.08–0.19]	— .897 <.001

In the univariate count analysis, both SSRI and SNRI use emerged as factors significantly associated with AF burden. SSRIs were associated with an approximately 70% reduction in days with AF (IRR 0.29 [0.09–0.95, *p* = .042), and SNRIs were associated with an approximately 90% reduction in the number of days with AF (IRR 0.09 [0.07–0.13], *p* < .001). Each 1 mL/m^2^ increase in indexed left atrial volume was associated with a 3% increase in the number of days in AF (IRR 1.03 [1.01–1.05], *p* = .003), and each 1% increase in left ventricular function was associated with a 3% reduction the number of days in AF in the number of days in AF (IRR 0.97 [0.96–0.99], *p* = .006). Of AF pharmacotherapies, sotalol use was associated with an approximately 80% decrease in the number of days in AF (IRR 0.22 [0.11–0.44], *p* < .001), and general beta blocker use was associated with a 6% decrease (IRR 0.94 [0.08–0.19], *p* < .001).

### 3.3 Multivariate regression

All estimates obtained from multivariate analysis are interpreted with the other covariates held at a constant level and summarized in Table 3. For the binary portion, results were similar to that in the univariate analysis. A yearly increase in age was associated with a 3% increase in the incidence of AF, (OR 1.03 [1.00–1.05], *p* = .027). Male, when compared with female gender, was associated with a reduction in AF incidence (OR 0.30 [0.13–0.68], *p* = .004).

**TABLE 3:** Multivariate regression coefficients for independent variables affecting AF burden.

Variable	Level	Odds ratio zero‐part (95% CI)	*p*‐value	Incidence rate ratio count part (95% CI)	*p*‐value
Antidepressant use	None SSRI SNRI Tetracyclic	1 .52 [0.13–2.18] 1.04 [0.12–8.94] 2.70 [0.33–22.1]	— .379 .972 .354	1 .27 [0.09–0.81] .07 [0.03–0.15] 1.10 [0.19–6.27]	— .019 <.001 .912
Age (years)		1.03 [1.00–1.05]	.027	1.00 [0.97–1.03]	.863
Sex	Female Male	1 0.30 [0.13–0.68]	— .004	1 1.31 [0.62–2.77]	— .486
Depression	No Yes	1 0.60 [0.18–2.00]	— .408	1 0.54 [0.18–1.65]	— .282
Anxiety	No Yes	1 2.00 [0.12–34.3]	— .630	1 1.64 [0.52–5.13]	— .397
BMI		1 1.00 [0.95–1.06]	— .907	1 1.04 [0.99–1.09]	— .131
LAVI		1 0.99 [0.97–1.02]	— .679	1 1.03 [1.01–1.05]	— .001
LVEF		1 0.98 [0.95–1.01]	— .164	1 0.95 [0.93–0.98]	— <.001
Amiodarone use	No Yes	1 0.87 [0.15–4.94]	— .871	1 0.93 [0.33–2.65]	— .896
Sotalol use	No Yes	1 2.01 [0.50–8.09]	— .325	1 0.30 [0.12–0.78]	— .013
Digoxin use	No Yes	1 4.43 [1.07–18.4]	— .04	1 2.51 [0.94–6.70]	— .065
Beta blocker use	None Heart failure beta blocker General beta blocker	1 0.94 [0.35–2.50] 2.31 [0.79–6.80]	— .915 .127	1 0.33 [0.14–0.81] 0.94 [0.30–2.92]	— .015 .917

After adjustment, digoxin remained significantly associated with the presence of AF (OR 4.43 [1.07–18.4], *p* = .04). Depression, anxiety, and antidepressant use were not significantly associated with the presence of AF. Similarly neutral results were obtained for BMI, LAVI, and LVEF.

In the count analysis, both SSRI and SNRI antidepressant use were associated with similar large reductions in days in AF, similar to the results of the univariate analysis (IRR 0.27 [0.09–0.81], *p* = .019 for SSRI and IRR 0.07 [0.03–0.15], *p* < .001 for both). Each 1 mL/m^2^ increase in left atrial diameter was still associated with a 3% increase in days in AF (IRR 1.03 [1.01–1.05], *p* < .001) and each 1% increase in LVEF was associated with a 5% reduction in days in AF (IRR 0.95 [0.93–0.98], *p* < .001). Sotalol still conferred a statistically significant decrease in AF burden (IRR 0.30 [0.12–0.78], *p* = .013). Heart failure beta blockade was associated with an approximately 70% decrease in AF burden (IRR 0.33 [0.14–0.81], *p* = .015).

## 4 DISCUSSION

In our cohort of older patients with CIEDs, a significant proportion of patients across both antidepressant use groups were noted to have AF, although there was no significant difference in AF burden between the cohorts at baseline. In our analysis, each yearly increase in age was associated with an increase in the odds of developing AF. This is consistent with the known increase in AF incidence with age.^14^ However, the lack of an association between age and AF burden possibly reflects a wide spectrum of AF severity in the elderly. The fact that male sex is associated with both lower AF incidence and burden is slightly surprising given that AF is more common in males,^15^ but may be related to a relatively older female population in our cohort.

Our results with BMI are contrary to expectations given that the literature has shown increases in AF incidence with increasing BMI.^16^ However, the associations between increasing LAVI and increasing AF burden is consistent with existing literature demonstrating increased LAVI as a marker of progression to permanent AF.^17^ With regards to the relationship between increasing LVEF and reduced burden of AF, this is consistent with the known bidirectional relationship of AF and heart failure, where neurohormonal mechanisms in worsening systolic dysfunction lead to increased remodeling in both ventricle and atrium.^18^

Heart failure‐specific beta‐blocker use, but not general‐beta blocker use, was associated with a decrease in AF burden. This may reflect a degree of reverse remodeling in heart failure with cardioselective beta‐blockade.^18^ The association between digoxin use and increased risk of odds of developing AF may reflect a degree of reverse causation as digoxin is often prescribed in sicker patients as a means of rate control for AF.^18^, ^19^

Sotalol is observed to be associated with a reduction in burden in AF in those already diagnosed, but has a non‐statistically significant association with increased incident AF. This is consistent with the prescription of sotalol to maintain normal rhythm after a limited episode of AF, which can either be self‐resolving or require medical intervention such as electrical cardioversion.^19^ Such effects may not have been seen with amiodarone, the other rhythm control agent evaluated in this study, due to prescription of amiodarone to patients with more severe left ventricular dysfunction (median LVEF 35% in patients on amiodarone versus 50% in those not prescribed amiodarone).

Despite population studies reporting an increase in incident AF^5^ with increasing depressive symptoms, our results are not suggestive of this, possibly reflecting differences in cohort size and comorbidity profile. Our results are however consistent with the lack of a strong association between anxiety disorders and incident AF as noted in the literature.^5^ The prevalence of these conditions in our study may also be underestimated as they may not be routinely entered into medical records, or they may undergo inconsistent coding.

Our data demonstrate that antidepressant use is not significantly associated with reduced odds of developing AF, but serotonin‐based antidepressants (SSRI and SNRIs) are both associated with a reduced number of days in AF. Traditionally, antidepressant use has been used as a surrogate indicator of depression in epidemiological studies^4^, ^7^; however, a large cohort study (*n* = 785 254) by Fenger‐Gron et al.^20^ demonstrate that the risk of incident AF is the highest in the month *prior* to commencing an antidepressant and progressively reduces with increasing time from commencement, suggesting a possible attenuating effect of antidepressants on AF. This study examined the effect of both SSRIs and SNRIs, but did not analyze them separately. It is unclear why serotonin‐based antidepressants are not associated with a significant reduction in the incidence of developing AF in our study. It may be the case that serotonin‐based antidepressants only have an effect on established cases of AF.

Lapi et al.^21^ in another large primary care cohort study, illustrated that the potency of serotonin inhibition at the 5HT‐4 receptor level by antidepressants was *not* associated with a reduction in chronic atrial fibrillation. Considering this, other mechanisms such as autonomic dysfunction emerge as a potential explanation for the proposed link between AF and anxious/depressive states.

Both arms of the autonomic nervous system seem to be involved in the triggering and propagation of AF. On one hand, reduced heart rate variability (an indicator of dysautonomia) in both general and AF populations is thought to be related to reduced parasympathetic tone.^22^ However, there is the observed phenomenon of vagally mediated AF in patients with structurally normal hearts,^23^ and evidence of increased parasympathetic innervation in animal models of AF.^24^ A recent randomized controlled trial by Wang et al showed that calcium paralysis of atrial ganglionic plexi during coronary artery bypass grafting reduced the incidence of post‐operative AF by about half (36% vs. 15%, HR 0.366 [0.211–0.635], *p* = .001).^25^ These plexi are situated near the pulmonary vein ostia (targets of catheter ablation for AF) and contain both sympathetic and parasympathetic nerve terminals.^26^

So far, there has been limited study into the impact of antidepressants on the autonomic nervous system. Only paroxetine, an SSRI, has been studied for its vagolytic effect and consequent reduction in AF episodes, but these results are limited to a case series of nine men.^27^ Several studies have found a reduction in HRV, and hence increased sympathetic tone, with antidepressant use in general,^12^, ^28^ and there remains some dispute over whether SSRIs may even increase HRV.^12^ However, given involvement of both sympathetic and parasympathetic nerves in AF,^24^ we postulate that perceived negative effects on HRV may overall be beneficial in the AF heart. Previously published data suggest serotonin activation at central 5HT‐2 receptors mediates a greater cardiovagal baroreflex response,^29^ and that SNRI users demonstrate increasing peripheral noradrenaline concentrations and hence sympathetic tone.^30^

The heterogeneity of information available underscores the need for further randomized controlled trials to assess the true impact of antidepressants on atrial arrhythmias.

### 4.1 Limitations

Due to the non‐randomized nature of the study, there may be unmeasured and unknown confounders that could not be accounted for by model adjustment. Furthermore, despite both manual and computer‐based matching of clinic visits to active medications, medication lists are usually based on a recent hospital or clinic summary letter and are not routinely collected on the day of the visit. Hospital documentation regarding the dose of medications is limited to inpatients, and most of our patients were outpatients in the clinic setting. Our results are related to an older population with cardiac implantable electronic devices and have limited generalizability to younger adults with atrial fibrillation.

To our knowledge, our study is the first to investigate the relationship between depression, anxiety, and AF using CIED‐recorded data. This provides a more long‐term, granular level of detail compared to existing studies that rely on binary responses of AF diagnoses from clinical records, and can quantify AF burden on a continuous scale, and is considered hypothesis‐generating.

## 5 CONCLUSION

In a study cohort of elderly patients with CIEDs, older age, larger left atrial volumes and reduced left ventricular ejection fraction were associated with a greater burden of AF. Serotonin‐based antidepressant use was associated with a significant reduction in AF burden. Further prospective studies to clarify the impact of antidepressants on atrial arrhythmias is warranted.

## AUTHOR CONTRIBUTIONS

Youlin Koh: study conception, data cleaning, and analysis, creation of first draft of manuscript. Cecilia Kwok: data collection, management, and analysis. Aleksandr Voskoboinik: study conception and design, data presentation, and further manuscript refinement. Jonathan M Kalman: study conception and design, data presentation and, further manuscript refinement. Michael Wong: study conception and design, data presentation and, further manuscript refinement.

## FUNDING INFORMATION

The authors acknowledge that this manuscript did not receive any external sources of funding.

## CONFLICT OF INTEREST STATEMENT

None declared.

## ETHICS APPROVAL AND PATIENT CONSENT STATEMENT

The study was approved by the Western Health Human Ethics Research Committee, who also granted a formal waiver of consent. De‐identification was performed by assignment of a subject ID and removal of hospital unit record numbers.

## Data Availability

Deidentified participant data are available upon reasonable request from Western Health, for the purposes of scientific replication. This is available by email to youlin.koh@wh.org.au.
